# Specific TLR-mediated HSP70 activation plays a potential role in host defense against the intestinal parasite *Giardia duodenalis*

**DOI:** 10.3389/fmicb.2023.1120048

**Published:** 2023-03-02

**Authors:** Min Liu, Yongwu Yang, Weining Zhu, Jingxue Wu, Xiran Yu, Wei Li

**Affiliations:** Heilongjiang Provincial Key Laboratory of Zoonosis, College of Veterinary Medicine, Northeast Agricultural University, Harbin, Heilongjiang, China

**Keywords:** *Giardia*, HSP70, host defense, apoptosis, nitric oxide, tight junction

## Abstract

*Giardia duodenalis*, an important flagellated noninvasive protozoan parasite, infects the upper small intestine and causes a disease termed giardiasis globally. Few members of the heat shock protein (HSP) family have been shown to function as potential defenders against microbial pathogens, while such information is lacking for *Giardia*. Here we initially screened and indicated that *in vitro Giardia* challenge induced a marked early upregulation of HSP70 in intestinal epithelial cells (IECs). As noted previously, apoptotic resistance, nitric oxide (NO)-dependent cytostatic effect and parasite clearance, and epithelial barrier integrity represent effective anti-*Giardia* host defense mechanisms. We then explored the function of HSP70 in modulating apoptosis, NO release, and tight junction (TJ) protein levels in *Giardia*-IEC interactions. HSP70 inhibition by quercetin promoted *Giardia*-induced IEC apoptosis, viability decrease, NO release reduction, and ZO-1 and occludin downregulation, while the agonist celastrol could reverse these *Giardia*-evoked effects. The results demonstrated that HSP70 played a previously unrecognized and important role in regulating anti-*Giardia* host defense *via* attenuating apoptosis, promoting cell survival, and maintaining NO and TJ levels. Owing to the significance of apoptotic resistance among those defense-related factors mentioned earlier, we then elucidated the anti-apoptotic mechanism of HSP70. It was evident that HSP70 could negatively regulate apoptosis in an intrinsic way *via* direct inhibition of Apaf-1 or ROS-Bax/Bcl-2-Apaf-1 axis, and in an extrinsic way *via* cIAP2-mediated inhibition of RIP1 activity. Most importantly, it was confirmed that HSP70 exerted its host defense function by downregulating apoptosis *via* Toll-like receptor 4 (TLR4) activation, upregulating NO release *via* TLR4/TLR2 activation, and upregulating TJ protein expression *via* TLR2 activation. HSP70 represented a checkpoint regulator providing the crucial link between specific TLR activation and anti-*Giardia* host defense responses. Strikingly, independent of the checkpoint role of HSP70, TLR4 activation was proven to downregulate TJ protein expression, and TLR2 activation to accelerate apoptosis. Altogether, this study identified HSP70 as a potentially vital defender against *Giardia*, and revealed its correlation with specific TLR activation. The clinical importance of HSP70 has been extensively demonstrated, while its role as an effective therapeutic target in human giardiasis remains elusive and thus needs to be further clarified.

## Introduction

*Giardia duodenalis* is a well-known zoonotic protozoan parasite responsible for over 280 million cases of gastrointestinal complaints annually across the world (Kraft et al., 2017). The life cycle of *Giardia* involves two stages: the noninvasive disease-causing vegetative form, trophozoite, and the environmentally resistant and infective form, cyst (Feng and Xiao, 2011; Vivancos et al., 2018). Cyst transmission occurs by the fecal-oral route, either by direct contact with *Giardia*-infected animals or by ingestion of cyst-contaminated soil, food, or water (Plutzer et al., 2010). *Giardia* infection impairs the integrity of the intestinal epithelial barrier and presents clinically as diarrhea, nausea, abdominal pain, vomiting, and weight loss (Vivancos et al., 2018). Up to now, little is known about the pathogenesis of giardiasis or the causes of post-giardiasis syndromes and treatment failures (Einarsson et al., 2016). It has been revealed that giardiasis development might be involved in the apoptotic outcome of intestinal epithelial cells (IECs), decreased nitric oxide (NO) production from IECs, and tight junction (TJ) disruption and barrier dysfunction (Eckmann, 2003; Lopez-Romero et al., 2015; Liu et al., 2018), whereas the underlying modulators remain to be illuminated.

Apoptosis operates as an important player in the pathogenesis of parasitic pathogens (Lüder et al., 2001; Benchimol and de Souza, 2022). Some parasites have evolved multiple strategies to trigger or block host cell apoptosis, so as to modulate host immune responses to facilitate dissemination within the host or maintain intracellular survival (Lüder et al., 2001). *Leishmania*-induced apoptosis is implicated in achievement of a prolonged parasite infection in macrophages (Real et al., 2014). *Trypanosoma cruzi* infection is causatively linked to high levels of host cell apoptosis, which dampens immune responses and favors parasite survival (Lopes et al., 2007). Extracellular *Giardia* infection can induce reactive oxygen species (ROS)-mediated mitochondrial and tumor necrosis factor receptor 1 (TNFR1)-mediated extrinsic pathways of apoptosis in IECs (Liu et al., 2020a,b). *Giardia*-induced IEC apoptosis can be one of the pathogenic mechanisms of giardiasis as described before (Troeger et al., 2007; Fisher et al., 2013). Cyclooxygenase-2 (COX-2)-mediated anti-IEC apoptosis represents a possible anti-*Giardia* host defense mechanism (Yang et al., 2022). NO is enzymatically produced from L-arginine and endogenously formed by three different NO synthase (NOS) isoforms: neuronal NOS (nNOS), inducible NOS (iNOS), and endothelial NOS (eNOS). iNOS is a major isoform induced in IECs by microbial products and cytokines and has a variety of biological functions (Eckmann, 2003; Muller and von Allmen, 2005). *Trypanosoma* infection can interfere with host immune responses by reducing the availability of arginine (Das et al., 2010; Chaturvedi et al., 2012). *Toxoplasma gondii* infection causes impaired defense-associated NO production by downregulating iNOS expression in macrophages (Cabral et al., 2018). NO derived from iNOS exerts an important function in limiting trophozoite growth and excystation process (Eckmann et al., 2000), but that derived from nNOS in promoting host clearance of *Giardia* (Li et al., 2006). However, the parasite has evolved strategies to evade this potential host defense by reducing epithelial NO production through competitive consumption of arginine as energy source (Eckmann et al., 2000; Ringqvist et al., 2008). In reality, downregulated iNOS expression is found in *Giardia*-infected IECs and paediatric patients (Mokrzycka et al., 2010; Stadelmann et al., 2013). *Giardia*-induced arginine depletion is responsible for the occurrence of IEC apoptosis (Ringqvist et al., 2008; Stadelmann et al., 2012). Strikingly, human giardiasis manifested as an increase in the apoptotic rate of IECs (Troeger et al., 2007; Fisher et al., 2013). Taken together, NO reduction induced by *Giardia* infection could be an indicator of giardiasis development. TJ is known as a major epithelial barrier maintained by TJ proteins like zonula occludens-1 (ZO-1), occludin, and claudins, which restricts paracellular permeability and balances the targeted transport and the exclusion of other unexpected pathogens (Buckley and Turner, 2018; Otani and Furuse, 2020). Although TJ serves as the front line of defense in impeding the entry of pathogenic microorganisms, some have evolved strategies that help reduce the entry barrier facilitating access to subepithelium and inner organs (Eichner et al., 2017). *Cryptosporidium parvum* infection leads to an enhancement of paracellular permeability across Caco-2 cell monolayers and a significant decrease in the levels of TJ proteins including ZO-1, occludin, and claudin-3/4 (Kumar et al., 2018). Noninvasive *Giardia* infection may lead to intestinal pathophysiology through disruption of tight junctional ZO-1 and increase of epithelial permeability (Buret et al., 2002). *Giardia*-expressed cysteine proteases are shown to disrupt IEC junctional complexes and barrier function (Liu et al., 2018). Therefore, to explore defense-related molecules and investigate their role in regulating giardiasis-involved factors is a crucial step to understand the complex regulatory network activated in *Giardia*-exposed IECs.

Heat shock proteins (HSPs) are constitutively expressed in all living organisms, some (HSP70, HSP90, HSP60/10, and sHSP) of which are induced as a response to diverse challenges and stresses and exhibit anti-apoptotic function, notably HSP70 (Zuo et al., 2016; Mayer, 2018; Abou-El-Naga, 2020). HSP70 is upregulated during *T. gondii* infection (Mitra et al., 2021). The elevated renal expressions of HSP70, iNOS, high mobility group protein B1, and nitrotyrosine appear in mice model of severe malaria (Fitri et al., 2017). HSP70 can bind directly to apoptotic protease activating factor-1 (Apaf-1) to block the recruitment of procaspase-9 to the apoptosome (Beere et al., 2000), and suppress bacterial toxin-induced mitochondrial ROS levels and preserve pulmonary microvascular endothelial barrier integrity (Li et al., 2018). HSP70 also has the capacity to block cell death process partially *via* stabilization of the E3 ubiquitin ligases of receptor-interacting protein 1 (RIP1) such as cellular inhibitor of apoptosis proteins (cIAPs) and X-linked inhibitor of apoptosis protein (XIAP; Srinivasan et al., 2018). HSP70 is required for iNOS induction in macrophages (Zhang et al., 2013), its externalization is able to upregulate NO production *via* Toll-like receptor 2 (TLR2) signaling (Song et al., 2013). HSP70 is also important in maintaining intestinal epithelial TJ barrier. HSP70-related heat preconditioning contributes vitally to the epithelial barrier recovery (Yang et al., 2007; Dokladny et al., 2008). This recovery can be accelerated in HSP70-overexpressing cells (Dokladny et al., 2006b; Yang et al., 2007), while impaired in cells expressing low levels of HSP70 (Musch et al., 1999; Dokladny et al., 2006a). Despite these advances, the role of HSP70 in defense-related apoptosis/NO/TJ regulation in the context of microbial pathogen infections remains largely unknown, notably *Giardia*. TLR functions as a critical linker between innate and adaptive immunity, and its activation is vital in host immune responses against microbial infections (Li et al., 2020). Our previous work has indicated that, during *Giardia*-IEC interactions, TLR4 activation gets involved in COX-2-mediated anti-apoptotic process and repression of NO downregulation (Yang et al., 2022). *Escherichia coli* MG1655 promotes TLR4/MyD88/p38 mitogen-activated protein kinase (MAPK) and endoplasmic reticulum stress (ERS) signaling-dependent intestinal epithelial injury and aggravates acute necrotizing pancreatitis in rats (Zheng et al., 2019). TLR2 has been indicated to be related to THP-1 cell apoptosis induced by *Aggregatibacter actinomycetemcomitans* (Kato et al., 2013). TLR2/6 signaling is specifically relevant to *T. cruzi* lipid-induced COX-2 expression and TNF-α and NO release in macrophages, as well as NF-κB activation and IL-8 release in HEK cells (Bott et al., 2018). *Lactobacillus acidophilus* leads to enhancement of intestinal epithelial TJ barrier and shows protection against intestinal inflammation in a strain-specific TLR2-dependent manner (Al-Sadi et al., 2021). TLR2 signaling on IECs involves enhancement of intestinal barrier function and prevention of deoxynivalenol-induced barrier dysfunction of epithelial cells (Gu et al., 2016). While it was noted above that TLR is responsible for various host responses, its involvement in HSP70 activation and HSP70-mediated defense responses against microbial infections remains to be elucidated. However, in certain circumstances, TLR4 has been shown to involve positive feedback regulation of HSP70 (Lee et al., 2013). It has been shown that HSP70 expression and extracellular release can be upregulated in macrophages by concurrent exposure to febrile range hyperthermia and agonists LPS for TLR4, Pam3Cys for TLR2, or Poly (I:C) for TLR3 (Gupta et al., 2013).

The aims of this study are to assess the functional relevance of HSP70 in defense responses against *Giardia* infection, majoring in anti-apoptosis and maintenance of NO levels and TJ barrier, as well as to investigate the role of HSP70 as a checkpoint regulator between specific TLR activation and defense initiation, for the purpose of fully understanding anti-*Giardia* host defense mechanism.

## Results

### *Giardia* challenge upregulated HSP70 expression in IECs

**Figure 1:**
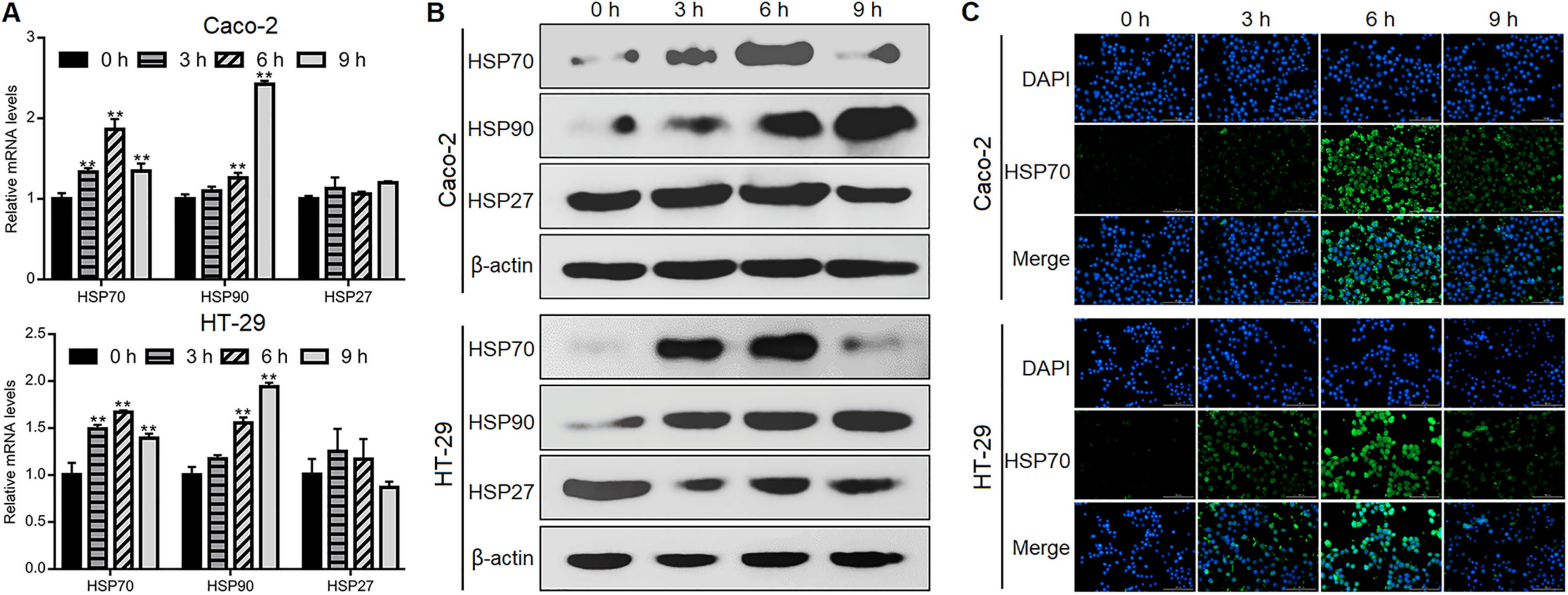
*Giardia* challenge upregulated HSP70 expression in IECs. Caco-2 and HT-29 cells were exposed to *Giardia* for 0, 3, 6, and 9 h. **(A–C)** The expression of HSPs was measured by qPCR and western blot analyses and immunofluorescence assay (scale bar = 100 μm). Data from triplicate wells from a representative of at least three independent experiments are presented as means ± SD. **p < 0.01.

We performed qPCR, western blotting, and immunofluorescence staining to investigate if *Giardia* could induce HSP activation in Caco-2 and HT-29 human intestinal cell lines. IECs were exposed with *Giardia* trophozoites at a ratio of 10 parasites/cell as noted before (Yang et al., 2022; Zhao et al., 2022), and the same applies hereinafter, although other ratios (5 or 20 parasites/cell) have been previously applied (Stadelmann et al., 2012; Amat et al., 2017). Upon challenge, the levels of mRNA transcription and protein expression of HSP70 and HSP90 were markedly increased in both Caco-2 and HT-29 cells within hours, while this is not the case for HSP27 (Figures 1A,B). In both cell types, HSP70 expression showed a marked upregulation at early time points and peaked at 6 h after *Giardia* exposure (Figures 1A–C). In contrast, HSP90 expression gradually increased in a time-dependent manner (Figures 1A,B). Taken together, among the candidate HSPs, HSP70 expression exhibited a marked early upregulation in IECs in response to infection with *Giardia*. On the basis of these observations, here HSP70 was selected as a target for analysis of its defense function.

### HSP70 regulated anti-apoptosis, cell survival, and NO levels

Our previous studies have shown that *Giardia* can induce Caco-2 cell apoptosis (Liu et al., 2020a,b), here we also confirmed the occurrence of apoptosis in *Giardia*-exposed HT-29 cells. Exposure of HT-29 cells with *Giardia* induced apoptosis as reflected by the appearance of an increasing amount of apoptotic cells stained orange by acridine orange (AO)/ethidium bromide (EB) double staining and a remarkable increase in the levels of cleaved caspase (CASP)-3 and cleaved poly(ADP-ribose) polymerase (PARP) by western blotting (Supplementary Figures 1A,B). *Giardia* exposure could also dramatically reduce the viability of HT-29 cells in a time-dependent manner as assessed by the CCK-8 assay (Supplementary Figure 1C). Additionally, NO release from HT-29 cells decreased time-dependently following *Giardia* exposure (Supplementary Figure 1D).

**Figure 2:**
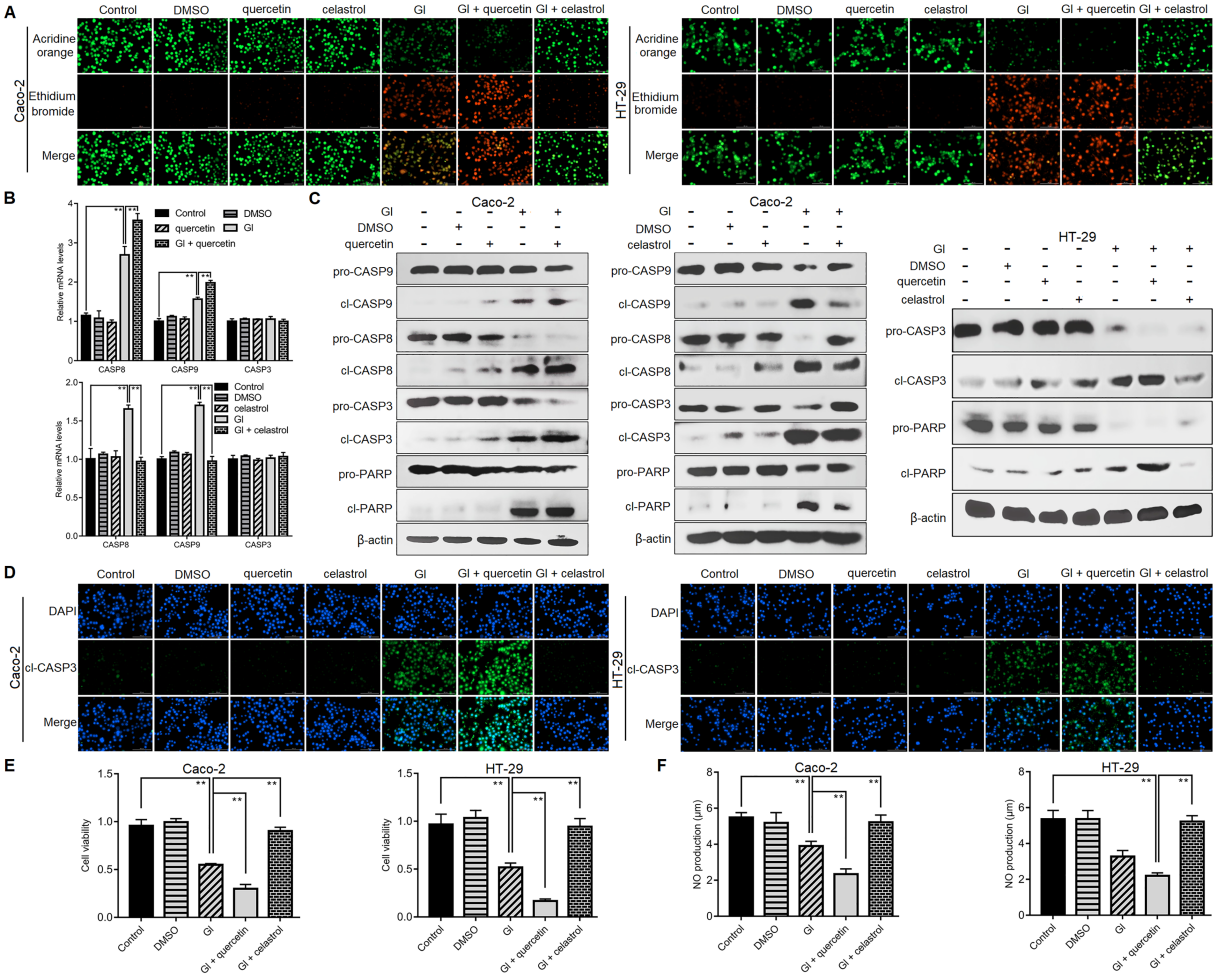
HSP70 regulated anti-apoptosis, cell survival, and NO levels. Prior to a 6-h exposure to *Giardia*, Caco-2 and HT-29 cells were treated with HSP70 antagonist, quercetin or agonist, celastrol. **(A)** HSP70 regulated anti-apoptosis as assessed by AO/EB staining (scale bar = 100 μm). **(B)** HSP70-mediated regulation of CASP-3/8/9 mRNA levels as measured by qPCR analysis. **(C)** HSP70-mediated regulation of protein levels of cleaved CASP-3/8/9 and PARP as measured by western blot analysis. **(D)** HSP70-mediated regulation of cleaved CASP-3 as examined using immunofluorescence assay (scale bar = 100 μm). **(E)** HSP70-mediated regulation of cell viability as measured by the CCK-8 assay. **(F)** HSP70-mediated NO regulation as examined by a microplate reader. Data from triplicate wells from a representative of at least three independent experiments are presented as means ± SD. **p < 0.01. Gl, *Giardia*.

We then explored the defense function of HSP70 in the context of *Giardia*-IEC interactions. While already recognized as an anti-apoptotic protein as introduced earlier, the role that HSP70 plays in *Giardia*-induced IEC apoptosis remains unclear. As reflected by the AO/EB assay, at 6 h after *Giardia* exposure, more obvious apoptosis-inducing effects were observed in Caco-2 and HT-29 cells when HSP70 inhibitor quercetin was applied (Figure 2A). In contrast, IEC apoptosis induced by a 6-h *Giardia* exposure could be blocked by preadministration of celastrol to overexpress HSP70 levels (Figure 2A). Moreover, in Caco-2 cells subjected to a 6-h exposure, HSP70 inhibition by quercetin could promote *Giardia*-induced upregulation of CASP-8 and CASP-9 at the mRNA levels (Figure 2B) and enhance the cleavage of CASP-8, CASP-9, CASP-3, and PARP (Figure 2C). By contrast, HSP70 overexpression by celastrol showed a notable inhibitory effect on Caco-2 cell apoptosis induced by a 6-h *Giardia* exposure (Figures 2B,C). The findings indicated that HSP70 impeded Caco-2 cell apoptosis probably *via* inhibition of *Giardia*-activated CASP-8/CASP-3-based extrinsic pathway (Liu et al., 2020b) and CASP-9/CASP-3-based intrinsic pathway (Liu et al., 2020a). HSP70 also exerted a function in promoting apoptotic resistance in HT-29 cells exposed with *Giardia* (Figure 2C). The anti-apoptotic function of HSP70 in IECs challenged with *Giardia* was further confirmed by immunofluorescence staining (Figure 2D). In the CCK-8 assays (Figure 2E), the use of HSP70 inhibitor quercetin reduced the viability of Caco-2 and HT-29 cells exposed to *Giardia* for 6 h, while the use of its agonist celastrol could rescue *Giardia*-decreased IEC viability. As shown in Figure 2F, *Giardia*-induced decrease of NO release in Caco-2 and HT-29 cells could be promoted by the application of quercetin and reversed by the application of celastrol. Collectively, our data revealed an essential role of HSP70 in protecting IECs against *Giardia* by regulating anti-apoptosis and cell survival and increasing NO release.

### HSP70 prevented *Giardia*-induced destruction of TJ proteins

**Figure 3:**
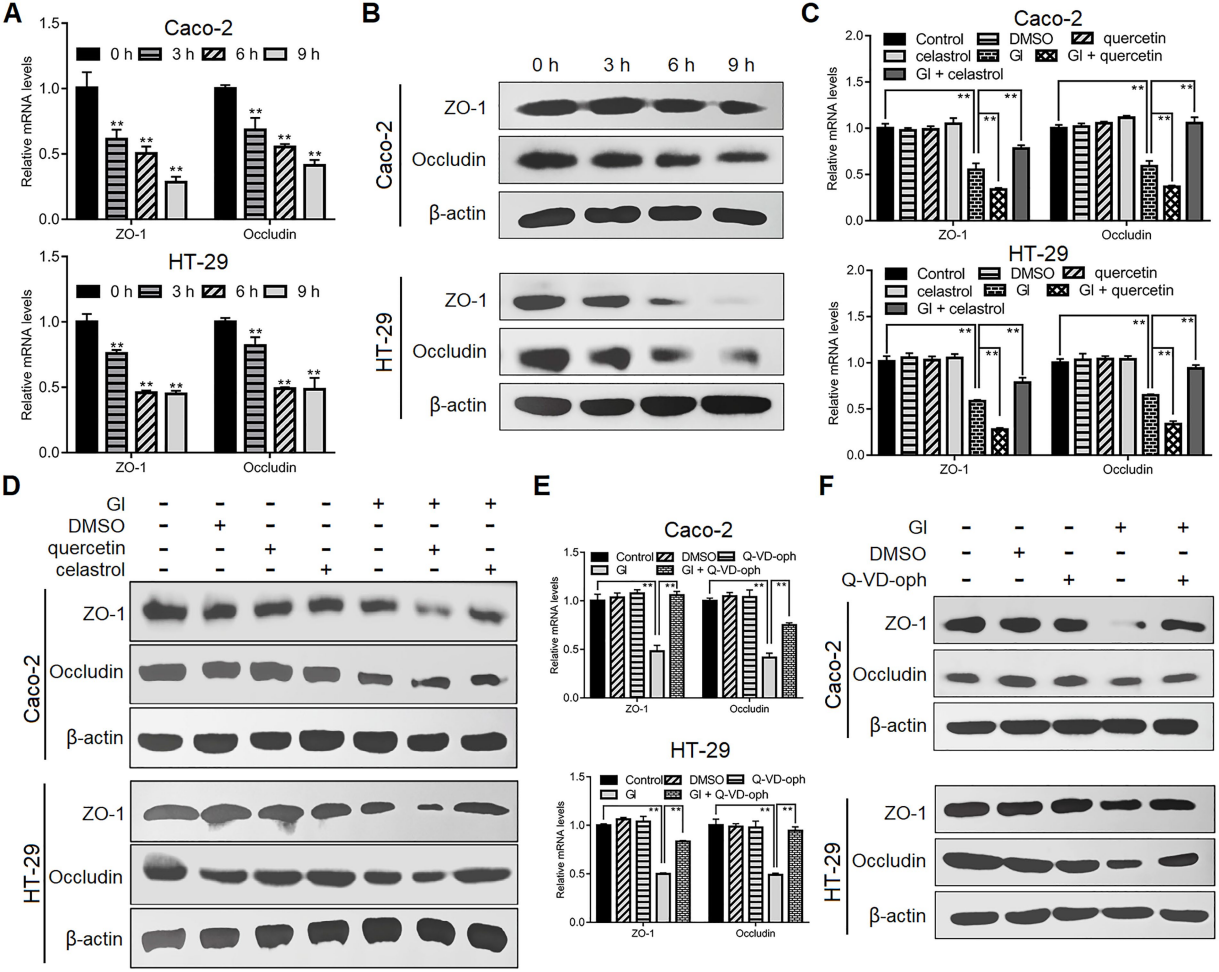
HSP70 prevented *Giardia*-induced destruction of TJ proteins. Unless otherwise specified, Caco-2 and HT-29 cells were exposed to *Giardia* for the indicated time periods. **(A,B)** *Giardia* downregulated ZO-1 and occludin following exposure as measured by qPCR and western blot. **(C,D)** In the case of application of quercetin or celastrol, HSP70 mediated reverse of decreased levels of ZO-1 and occludin induced by a 6-h *Giardia* exposure as measured by qPCR and western blot. **(E,F)** Prior to a 6-h exposure to *Giardia*, cells were incubated with a pan-caspase inhibitor Q-VD-OPh. *Giardia*-induced IEC apoptosis modulated the levels of ZO-1 and occludin as measured by qPCR and western blot. Data from triplicate wells from a representative of at least three independent experiments are presented as means ± SD. **p < 0.01. Gl, *Giardia*.

As noted earlier, TJ proteins are important for epithelial barrier function related to anti-*Giardia* host defense mechanism. Following *Giardia* challenge, ZO-1 and occludin expressions were markedly decreased at both mRNA and protein levels in Caco-2 and HT-29 cells (Figures 3A,B). Moreover, such process induced by a 6-h *Giardia* exposure could be exacerbated by inhibition of HSP70 with quercetin and blocked by overexpression of HSP70 with celastrol (Figures 3C,D). In addition, interestingly, preincubation of IECs with a pan-caspase inhibitor, Q-VD-OPh could reverse *Giardia*-induced downregulation of ZO-1 and occludin expressions (Figures 3E,F). In light of the established role of HSP70 in apoptosis/TJ regulation, we could infer that HSP70 acted as a promoter for maintaining TJ barrier integrity *via* inhibiting apoptosis during *Giardia*-IEC interactions.

### The anti-apoptotic mechanism of HSP70 *via* intrinsic and extrinsic pathways

**Figure 4:**
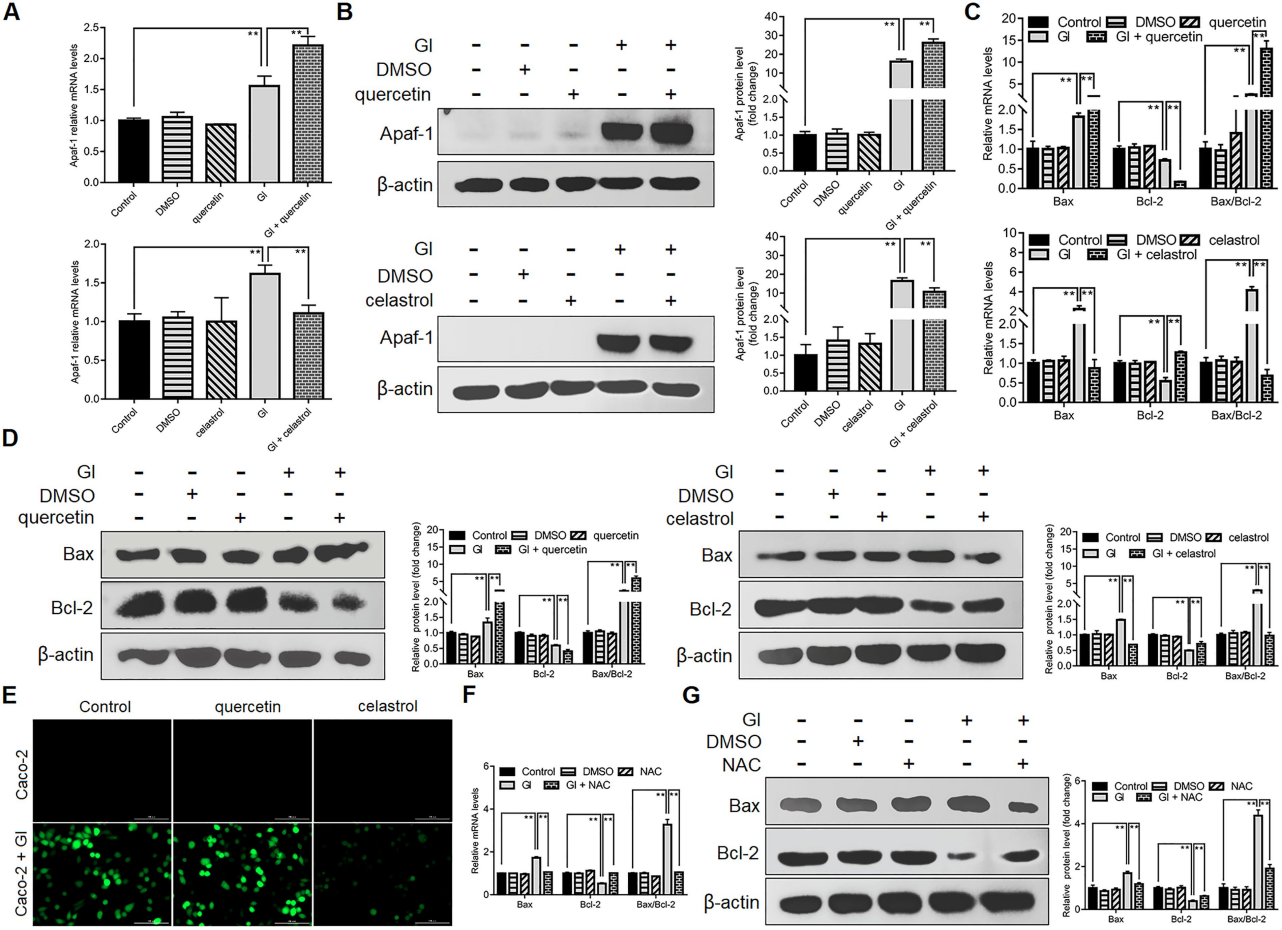
Anti-apoptotic mechanism of HSP70 in an intrinsic way. Prior to a 6-h exposure to *Giardia*, Caco-2 cells were treated with quercetin or celastrol. **(A,B)** HSP70-mediated regulation of Apaf-1 as measured by qPCR and western blot. **(C,D)** HSP70-mediated regulation of the Bax/Bcl-2 ratio as measured by qPCR and western blot. **(E)** HSP70-mediated ROS regulation as examined by immunofluorescence assay (scale bar = 100 μm). **(F,G)** ROS-mediated regulation of the Bax/Bcl-2 ratio as measured by qPCR and western blot. Data from triplicate wells from a representative of at least three independent experiments are presented as means ± SD. **p < 0.01. Gl, *Giardia*.

Anti-apoptosis is outstanding among the HSP70-regulated defense-related factors since NO and TJ levels are tightly related to the apoptotic outcomes of IECs induced by *Giardia*. It was also shown earlier that HSP70 could operate as an effective player in preventing Caco-2 cell apoptosis *via* intrinsic and extrinsic pathways, while the underlying mechanism is not clear. *Giardia* is capable of inducing Caco-2 cell apoptosis *via* ROS-mediated intrinsic or mitochondrial pathway by modulating the ratio of Bcl-2-associated X (Bax) protein to B-cell lymphoma-2 (Bcl-2) protein and the levels of cleaved apoptotic initiator CASP-9 and effector CASP-3 as noted (Liu et al., 2020a). Here HSP70 inhibition by quercetin could promote *Giardia*-induced upregulation of Apaf-1 at both mRNA and protein levels in Caco-2 cells, while its overexpression by celastrol could reverse this process (Figures 4A,B), indicating an inhibitory effect of HSP70 on *Giardia*-activated proapoptotic factor Apaf-1. It has been proved that direct binding of HSP70 to Apaf-1 can inhibit the interaction between Apaf-1 and CASP-9 (Beere et al., 2000). Given the role of HSP70 in impeding CASP-9/3-dependent apoptosis demonstrated earlier, it seemed likely that HSP70 can inhibit *Giardia*-induced intrinsic apoptosis *via* interacting directly with Apaf-1 and thus blocking CASP-9 activation. In addition, yet, Bcl-2 can prevent Bax-activated release of cytochrome c (Cyt-c) from mitochondria and therefore block Cyt-c from promoting Apaf-1-mediated CASP-9 activation (Zou et al., 1999), leading to another hypothesis that HSP70 prevents *Giardia*-initiated Apaf-1 activation through regulating Bcl-2 and Bcl-2-associated X protein, Bax. As expected, here we confirmed that HSP70 participated in negatively regulating the Bax/Bcl-2 ratio (Figures 4C,D). We further identified the negative role of HSP70 in regulating ROS levels (Figure 4E) and the positive role of ROS in regulating the Bax/Bcl-2 ratio (Figures 4F,G). Taken together, it is not difficult to infer that HSP70 prevented *Giardia*-induced Caco-2 cell apoptosis in an intrinsic way *via* ROS-Bax/Bcl-2-Apaf-1 axis as well.

**Figure 5:**
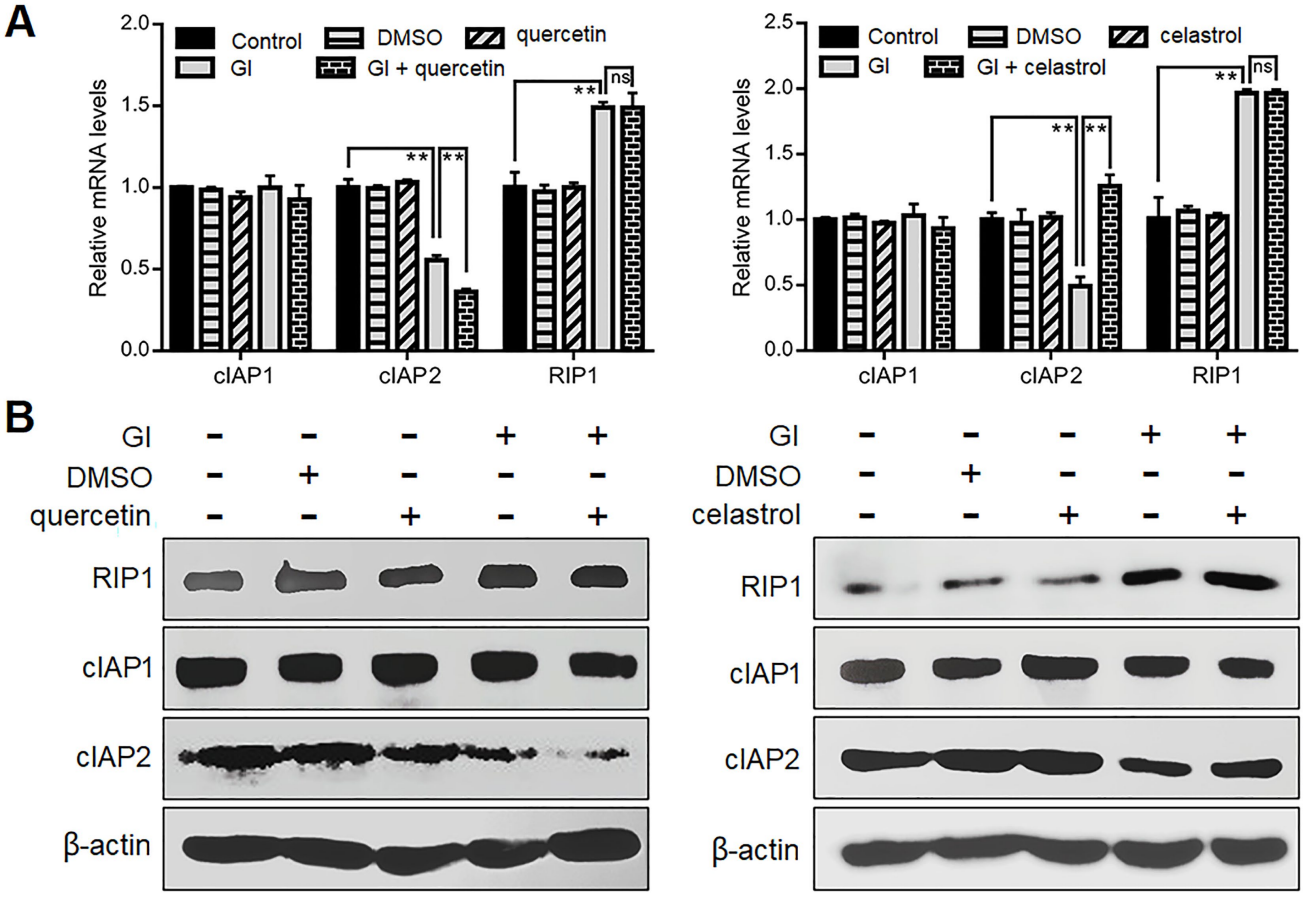
Anti-apoptotic mechanism of HSP70 in an extrinsic way. Prior to a 6-h exposure to *Giardia*, Caco-2 cells were treated with quercetin or celastrol. **(A,B)** HSP70-mediated regulation of cIAP1/2 and RIP1 as assessed by qPCR and western blot. Data from triplicate wells from a representative of at least three independent experiments are presented as means ± SD. **p < 0.01. Gl, *Giardia*; ns, not significant.

It has also been noted that *Giardia* can induce CASP-8/CASP-3-depedent extrinsic pathway of apoptosis in Caco-2 cells *via* TNFR1 activation and RIP1 deubiquitination (Liu et al., 2020b). The ubiquitination status of RIP1 mediated by its E3 ubiquitin ligases cIAP1/2 is critical for the transition of signaling complex I (activating NF-κB/MAPK signaling to promote cell survival) to complex IIa (activating CASP-8 to initiate apoptosis) (Zhang et al., 2019). In addition, the prevention of RIP1-dependent cell death may involve HSP70-mediated stabilization of cIAPs in cancer cells (Srinivasan et al., 2018). From those observations, we assumed here that HSP70 affects the ubiquitination status of RIP1 *via* stabilizing its regulator cIAP1/2 during *Giardia*-IEC interactions. There were no observed changes in the cIAP1 expression between *Giardia*-exposed and -unexposed groups at both mRNA and protein levels (Figures 5A,B). Nevertheless, *Giardia* challenge led to a remarkable decrease in the mRNA and protein levels of cIAP2, and this process could be accelerated by the use of quercetin and reversed by the use of celastrol (Figures 5A,B), indicative of involvement of HSP70 in the stability of cIAP2. In addition, a significant increase in the RIP1 expression was observed following *Giardia* challenge, while the elevation could not be affected by HSP70 inhibition or overexpression (Figures 5A,B). Collectively, it could be inferred that HSP70 prevented *Giardia*-induced CASP-8/CASP-3-dependent extrinsic apoptosis probably through maintaining native levels of cIAP2 that is important for RIP1 ubiquitination.

### TLR4 involved HSP70-mediated defense-related apoptosis and NO regulation

**Figure 6:**
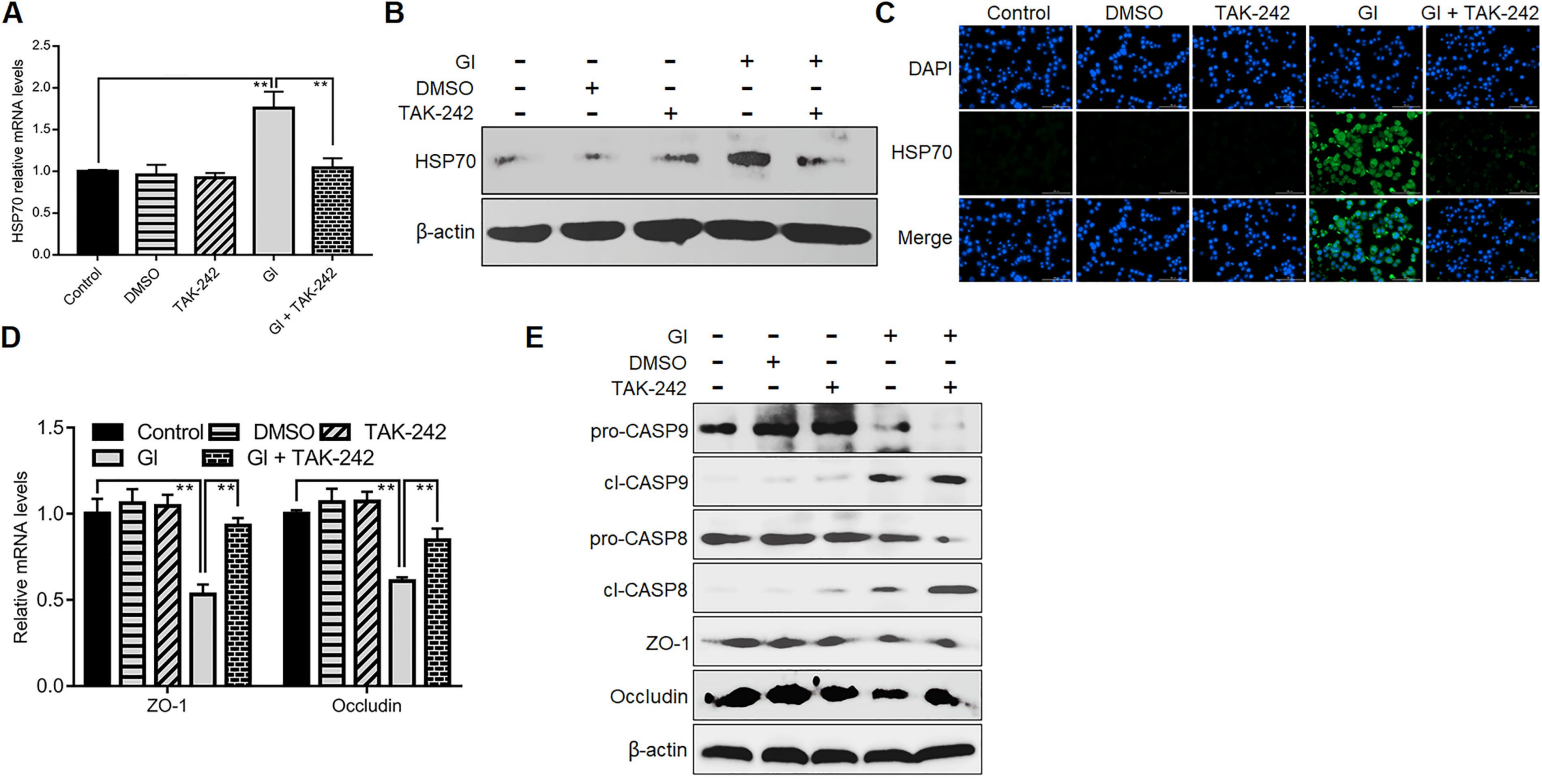
TLR4 involved HSP70-mediated defense-related apoptosis and NO regulation. Prior to a 6-h exposure with *Giardia*, Caco-2 cells were administrated with TLR4 antagonist TAK-242. **(A–C)** TLR4-mediated HSP70 regulation as measured by qPCR, western blot, and immunofluorescence analyses (scale bar = 100 μm). **(D)** TLR4-mediated regulation of ZO-1 and occludin at the mRNA levels as measured by qPCR analysis. **(E)** TLR4-mediated regulation of the levels of ZO-1, occludin, and cleaved CASP-8/9 as assayed by western blot. Data from triplicate wells from a representative of at least three independent experiments are presented as means ± SD. **p < 0.01. Gl, *Giardia*.

We further evaluated the association between TLR4 signaling and HSP70-mediated anti-*Giardia* host defense responses. TLR4 inhibition by its antagonist TAK-242 suppressed upregulation of HSP70 in Caco-2 cells induced by a 6-h *Giardia* exposure (Figures 6A–C), suggesting a positive regulatory relationship between TLR4 and HSP70. TLR4 activation has been shown to protect Caco-2 cells against *Giardia*-induced apoptosis and NO reduction (Yang et al., 2022). Given the role of HSP70 in preventing *Giardia*-induced apoptosis and NO reduction in IECs established earlier here, it could be concluded that TLR4 activation correlated positively to HSP70-mediated anti-apoptosis and recovery of NO levels during *Giardia*-IEC interactions. However, this would not be the case for TJ proteins ZO-1 and occludin which could be positively regulated by HSP70 as we validated earlier, while negatively regulated by TLR4 as we observed currently (Figures 6D,E). In addition, like HSP70, TLR4 could also mediate inhibition of CASP-9 and CASP-8 activation (Figure 6E), expanding the HSP70-mediated anti-apoptotic signaling network during *Giardia* infections.

### TLR2 involved HSP70-mediated defense-related TJ and NO regulation

**Figure 7:**
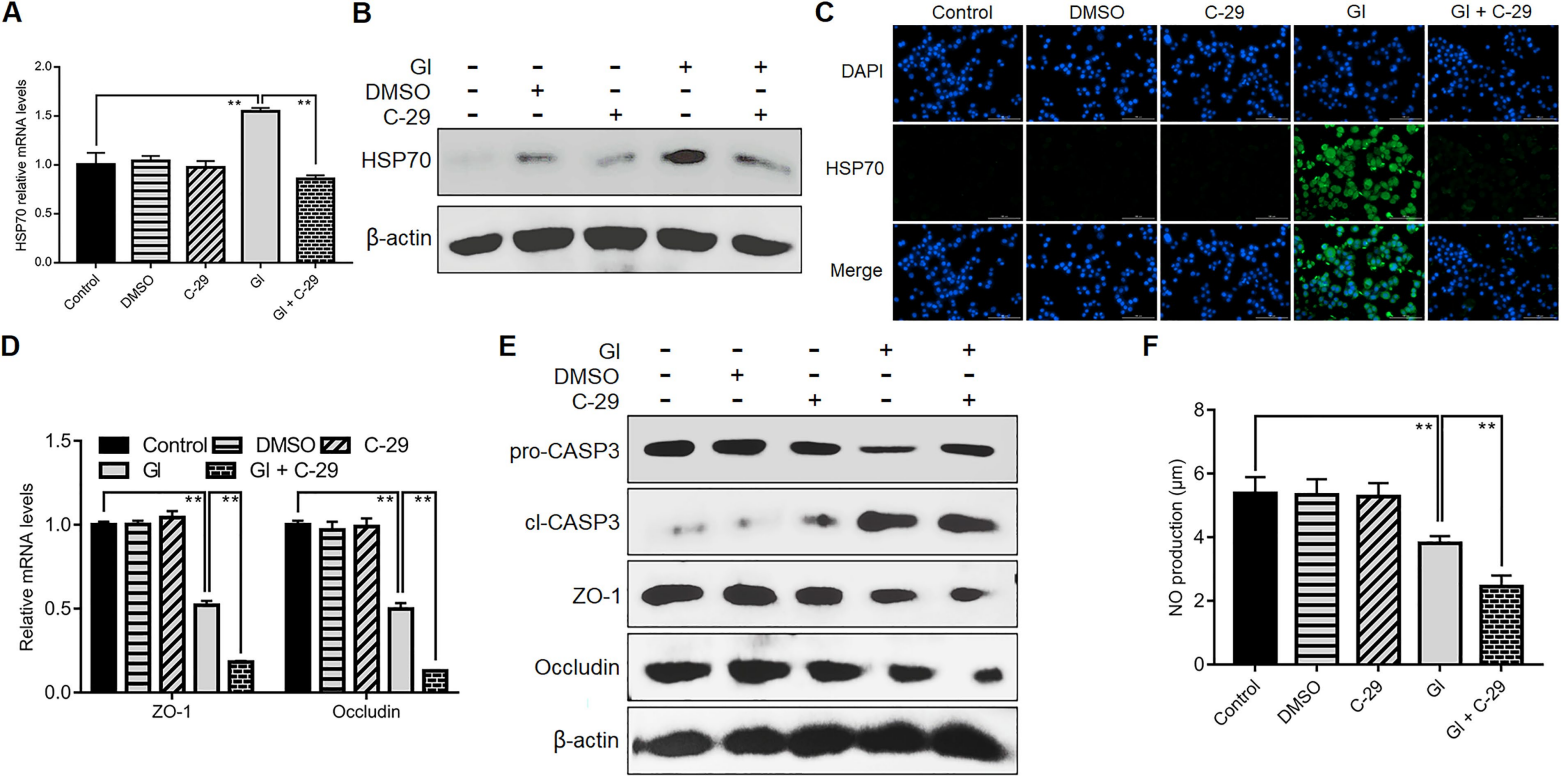
TLR2 involved HSP70-mediated defense-related TJ and NO regulation. Prior to a 6-h exposure to *Giardia*, Caco-2 cells were incubated with TLR2 inhibitor C-29. **(A–C)** TLR2-mediated HSP70 regulation as measured by qPCR, western blot, and immunofluorescence analyses (scale bar = 100 μm). **(D)** TLR2-mediated regulation of ZO-1 and occludin at the mRNA levels as measured by qPCR analysis. **(E)** TLR2-mediated regulation of the levels of ZO-1, occludin, and cleaved CASP-3 as assayed by western blot. **(F)** TLR2-mediated NO regulation as measured by a microplate reader. Data from triplicate wells from a representative of at least three independent experiments are presented as means ± SD. **p < 0.01. Gl, *Giardia*.

TLR2 would function in HSP70-mediated anti-*Giardia* host defense responses in a manner different from TLR4. Like TLR4, during the interactions between *Giardia* and Caco-2 cells, there was a positive correlation between TLR2 and HSP70 as validated by application of TLR2 antagonist C-29 (Figures 7A–C). Unlike TLR4, TLR2 operated as a player in positive regulation of TJ proteins ZO-1 and occludin (Figures 7D,E). In addition, TLR2 activation impeded *Giardia*-induced NO reduction (Figure 7F). Given the known positive associations between HSP70 and TJ/NO, it can be concluded that TLR2 was positively related to HSP70-mediated maintenance of TJ barrier and NO levels. Yet, unlike TLR4, TLR2 served as an apoptosis promoter during *Giardia* infections (Figure 7F).

## Discussion

**Figure 8:**
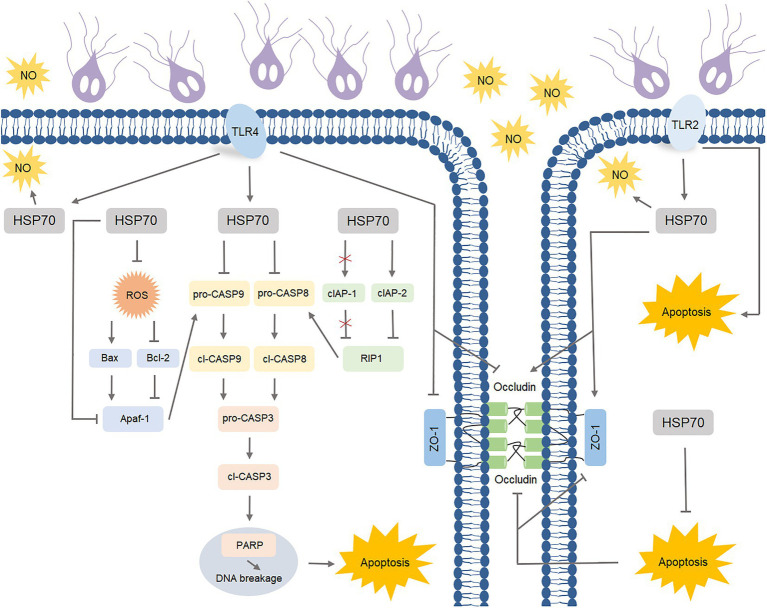
Schematic diagram illustrating TLR-activated HSP70-mediated defense signaling network during *Giardia*-IEC interactions.

*Giardia* is a zoonotic intestinal parasite and a leading cause of diarrheal disease worldwide (Ankarklev et al., 2010). Thus far, it remains largely unknown about the effective players in the complex defense-signaling network of IECs in response to *Giardia* infection. In the resent study, we initially acknowledged the role of HSP70 in defending against *Giardia via* preventing IEC apoptosis and maintaining the levels of NO and TJ proteins. The intrinsic and extrinsic anti-apoptotic mechanisms of HSP70 were subsequently explored. Specific TLR activation was then linked to HSP70-mediated anti-*Giardia* host defense responses (Figure 8).

HSP70 is a highly evolutionarily conserved chaperone that is overexpressed in the cells in response to stress of various origins including infection (Tukaj, 2020). HSP70 expression is elevated following *T. gondii* infection (Mitra et al., 2021) and malaria infection (Fitri et al., 2017). In this study, noninvasive *Giardia* also evoked a rapid HSP70-mediated defense response similar to that caused by those invasive protozoan parasites. HSP70 is known as a robust anti-apoptotic protein involving a wide number of cell survival aspects (Abou-El-Naga, 2020), while the role it plays in microbial infections remains largely unexplored. Herein, HSP70 was identified as an effective anti-apoptotic operator in *Giardia*-infected IECs *via* regulating intrinsic (ROS, Bax, Bcl-2, Apaf-1, and CASP-9) and extrinsic (cIAP1, RIP1, and CASP-8) factors identified in our previous studies (Liu et al., 2020a,b), which could promote cell survival as measured by CCK-8 assay and hence contribute to host defense mechanism. *Giardia* has been documented to inhibit NO production in IECs by competitive consumption of local arginine and low arginine levels might exacerbate IEC apoptosis (Eckmann et al., 2000), meanwhile, NO of various origins represents an important *Giardia*-growth inhibitor as well as an inducer for intestinal peristalsis, helping discharge the parasite from the intestine (Eckmann et al., 2000; Li et al., 2006). Considering this situation, in this study, HSP70-regulated recovery of NO levels could be an important part of the host defense in *Giardia* elimination. It has been acknowledged that adhesion of *Giardia* trophozoites to IECs induces disturbances of intercellular junctions (tight, adherens, and desmosomal junctions) (Maia-Brigagao et al., 2012). Additionally, *Giardia*-induced IEC apoptosis contributes to the development of giardiasis *via* accelerating normal turnover of intestinal epithelium and disrupting epithelial barrier function (Panaro et al., 2007). Here we confirmed that HSP70 functioned as a positive regulator for TJ proteins, in part through inhibiting IEC apoptosis. Enhanced integrity of intestinal epithelial barrier mediated by HSP70 could favor anti-*Giardia* host defense. The collective data supported a crucial role of HSP70 in modulating anti-*Giardia* defense responses by augmenting anti-apoptosis of IECs and maintaining NO and TJ levels. HSP70 has gained increasing attention as a therapeutic target for many diseases such as stroke and other related neurological conditions (Kim et al., 2018). HSP70 blocking has clinical implications in cancer therapy partially *via* activating a specific anticancer immune response (Goloudina et al., 2012). Thus far, *Giardia* infection is still a worldwide public health problem that poses a serious threat to human health (Dixon, 2021; Mahdavi et al., 2022). Therefore, more efforts need to be devoted towards investigating the role of HSP70 as a therapeutic target for human giardiasis. In addition, it is of interest to note that HSP70 is also present in *Giardia* (Gupta et al., 1994; Arisue et al., 2002), which it is possibly involved in the increased levels of the HSP70 protein in the serum of *Giardia*-infected individuals (Al-Madani and Al-Khuzaie, 2021).

TLRs are a well-known family of pattern recognition receptors in innate immunity that recognize and defend invasive and noninvasive microbial pathogens (Liu et al., 2014). The various TLRs mediate different cellular responses to pathogens due to particular usage of intracellular adapter proteins (Kawai and Akira, 2007). TLR4 serves an important function in host responses to tissue injury, microbial infection, and inflammation (Kawai and Akira, 2007). *Giardia*-activated TLR4 signaling has been shown to involve COX-2-mediated blocking of apoptosis and NO reduction (Yang et al., 2022). Here TLR4 signaling was specifically linked to HSP70-initiated anti-*Giardia* defense responses through impeding apoptosis and NO reduction. TLR2 is another member of the TLR family that plays a fundamental role in pathogen recognition and activation of innate immunity (Kawai and Akira, 2007). TLR2 is reported to regulate the initial pro-inflammatory response during infection of macrophages with *T. cruzi* (Ropert and Gazzinelli, 2004). Mast cells can phagocyte *Candida albicans* and generate NO by the mechanisms related to TLR2 and Dectin-1 (Pinke et al., 2016). In the present study, we indicated that TLR2-dependent HSP70 activation was able to reverse *Giardia*-decreased NO levels, which agreed with what we observed in TLR4 regulation. In addition, TLR2 activation has been proven as an important part of the response to murine and human skin barrier repair through enhancing TJ barrier and expression of TJ proteins such as ZO-1, occludin, and claudin-1/23 (Kuo et al., 2013). Here *Giardia*-activated TLR2-HSP70 signaling was responsible for maintaining the levels of TJ proteins ZO-1 and occludin, potentially contributing to the integrity of intestinal epithelial barrier that is important for the host defense against the parasite. However, this finding was contrary to the negative role of TLR4 in TJ regulation established earlier in this study. In reality, TLR4 activation appears to involve LPS-induced increase in intestinal TJ permeability (Guo et al., 2013). It is also noteworthy that, unlike TLR4, TLR2 could mediate a proapoptotic response in *Giardia*-exposed IECs. Actually, TLR2 and neutrophils have been described to potentiate endothelial ER stress, apoptosis, and detachment (Quillard et al., 2015).

## Conclusion

Here we demonstrated a potential role of HSP70 in mediating host defense responses against *Giardia* using *in vitro* culture models. The underlying regulatory network was complex and involves multiple factors. HSP70 function and *Giardia* evasion of this function may contribute significantly to the balance between host defense and giardiasis progression. Our study also indicated a close link between specific TLR activation and HSP70-mediated defense responses. Taken together, the results of this study provided further detailed insights into the interactions between *Giardia* and IECs in terms of host defense and pathogenesis. Although sound, substantial additional efforts are required to characterize more defense-related modulators and probe their correlations with giardiasis-associated dysregulations, which is central for introducing novel and effective therapeutics in the clinics. However, in any case, the *in vitro* interaction system used in this study could not present complexity of interactions from neighboring cells of different types and functions. It is therefore essential to use organoid-derived monolayers and/or an accessible laboratory animal model to fully elucidate the role of specific TLR-mediated HSP70 activation in anti-*Giardia* host defense responses.

## Materials and methods

### Study design

This study was designed to investigate the role of HSP70 and the related molecular and cellular mechanisms involved in host defense against noninvasive *Giardia* infection by the use of an *in vitro* model of the interaction between human-derived *Giardia* trophozoites and Caco-2/HT-29 cell lines as previously described (Kleiveland, 2015; Emery et al., 2016; Ma’ayeh et al., 2017; Dubourg et al., 2018; Liu et al., 2020a,b; Yang et al., 2022).

### Cell culture

The human colon adenocarcinoma cell lines Caco-2 and HT-29 that closely resemble normal human small IECs (Martinez-Maqueda et al., 2015; Estermann et al., 2020), were purchased from the Cell Bank of the Chinese Academy of Sciences (Shanghai, China) and used to interact with *Giardia* trophozoites *in vitro*. Caco-2 cell line was grown in high-glucose DMEM (Hyclone, Logan, United States) supplemented with 10% FBS, 1% MEM NEAA, 1% GlutaMAX, and 1% penicillin/streptomycin. By contrast, HT-29 cells were grown in DMEM/F12 (Hyclone, Logan, United States) supplemented with 10% FBS and 1% penicillin/streptomycin. The two types of cell lines were incubated at 37°C and 5% CO_2_, and sub-cultured every three to 4 days at 80–90% confluence before being seeded. Culture medium was changed every second day. Cells were seeded in 6-well (1 × 10^6^ cells/well), 12-well (5 × 10^5^ cells/well), 24-well (2 × 10^5^ cells/well), and 96-well (1 × 10^4^ cells/well) plates depending on the needs of the experiments. All experiments were performed 2–3 days post-seeding at 80–90% confluence, except those regarding evaluation of TJ protein levels which were performed 2–3 days post-confluence on fully differentiated, confluent monolayers.

### Parasite culture and exposure

The human-derived *G. duodenalis* WB isolate (genotyped as zoonotic assemblage A, ATCC 30957, Manassas, United States) was applied in this study. Trophozoites were propagated at 37°C in 15 ml conical bottomed tubes in modified TYI-S-33 medium containing 10% FBS and 0.1% bovine bile supplemented with 0.1% gentamicin and 1% penicillin/streptomycin (Keister, 1983). Cultures were harvested by chilling on ice for 15 min. Detached trophozoites were centrifuged, washed with PBS, resuspended in cell culture medium, counted using a hematocytometer, and used to challenge cells at a ratio of 10 parasites/cell. Prior to challenge, an endotoxin ELISA kit (Meimian Biotech, Yancheng, China) was applied to ensure that the washed parasites and resuspension medium were free of endotoxins that would be responsible for the observed TLR activation. In time-course experiments, trophozoites were added at different time points and cells harvested together. Prior to further analysis, cells were rinsed thrice with ice-cold PBS to remove parasites.

### Protein inhibition and overexpression

We used HSP70 inhibitor quercetin (20 μM in use; AbMole, Houston, United States), ROS inhibitor NAC (10 μM; APEXBIO, Houston, United States), pan-caspase inhibitor Q-VD-OPh (50 μM; AbMole, Houston, United States), TLR4 inhibitor TAK-242 (10 μM; APEXBIO, Houston, United States), and TLR2 inhibitor C-29 (20 μM, APEXBIO, Houston, United States) in inhibition analyses. HSP70 inhibitor quercetin was applied 2 h before exposure, while other inhibitors were applied 1 h before *Giardia* exposure. A 0.1% DMSO solution was used as the solvent control. Overexpression of HSP70 was conducted using celastrol (AbMole, Houston, United States) at the concentration of 3 μM. The agonist was applied 30 min before *Giardia* exposure. A 0.1% DMSO solution was used as the solvent control as well. Before further analysis, cells were rinsed thrice with PBS for drug removal.

### qPCR analysis

Cells cultured in 12-well plates were harvested for RNA extraction using Trizol reagent (Invitrogen, Carlsbad, United States). cDNA was synthesized from total RNA (1 μg) using a HiScript II 1st Strand cDNA Synthesis Kit (Vazyme, Nanjing, China). Amplification was conducted using a SYBR-Green qPCR Master Mix Kit (Vazyme, Nanjing, China) on a LC480 Lightcycler system (Roche, Indianapolis, United States). Primer pairs used in qPCR analysis were shown in Supplementary Table 1. The expression of target genes relative to the housekeeping gene, GAPDH, was analyzed using the 2^−ΔΔ*C*t^ method.

### Western blot analysis

Cells cultured in 6-well plates were harvested for protein extraction using RIPA lysis buffer (Beyotime, Shanghai, China) containing 1% PMSF (Beyotime, Shanghai, China). Total protein concentration was measured by an enhanced BCA Protein Assay Kit (Beyotime, Shanghai, China). The extracted proteins were separated by 12% SDS-PAGE and transferred to PVDF membranes by electro blotting. Membranes were blocked with 5% skim milk in PBST at room temperature (RT) for 2 h and then incubated at 4°C for 12 h with the primary antibodies (1:1,000 dilution in PBST) against HSP70, HSP90, HSP27, pro-/cl-CASP-3, pro-/cl-PARP, pro-/cl-CASP-9, pro-/cl-CASP-8, Apaf-1, Bax, Bcl-2, RIP1, cIAP1/2, ZO-1, occludin, and β-actin. Primary antibodies were obtained from two commercial sources (ABclonal, Wuhan, China; Bioss, Beijing, China). Membranes were washed three times for about 1 h in PBST and then probed with HRP-conjugated secondary antibody (1:5,000 dilution in PBST; ABMART, Shanghai, China) at RT for 1 h. Images were obtained from a GeneGnome XRQ chemiluminescence imaging system (Syngene, Cambridge, United Kingdom), and the intensity of the detected bands was quantified with the NIH Image J software (NIH, Bethesda, United States).

### Immunofluorescence assay

Cells grown on cover slips in 24-well plates were fixed with 4% paraformaldehyde at RT for 30 min, and permeabilized with 0.25% Triton-X 100 at RT for 10 min. Nonspecific binding sites were blocked by incubation in 1% BSA in PBS at RT for 1 h. Cells were incubated with the primary antibodies against HSP70 (dilution, 1:300) and cl-CASP-3 (dilution, 1:100) at 4°C overnight, and then FITC-AffiniPure Goat Anti-Rabbit IgG (H + L) (dilution, 1:200; Jackson, West Grove, United States) at 37°C for 1 h protected from the light. Cell nucleus was stained by 2 μg/ml DAPI (Alphabio, Tianjin, China). Images were captured by a Lionheart FX Automated Microscope (BioTek, Winooski, United States).

### AO/EB staining

Cells grown on cover slips in 24-well plates were evaluated for *Giardia*-induced IEC apoptosis by dual staining with combined fluorescent dyes AO and EB (BestBio, Shanghai, China) and observed by using a Lionheart FX Automated Microscope. Normal cells were stained green and apoptotic cells were stained orange as previously described (Gherghi et al., 2003).

### Cell viability assay

Prior to drug or *Giardia* exposure, an additional incubation of cells in FBS-free medium in 96-well plates for 3 h was needed in this part. Cell viability was measured by a CCK-8 assay (Apexbio, Houston, United States) as described (Cai et al., 2021), and negative control wells containing just DMEM and trophozoites in DMEM were included. The absorbance was measured at 450 nm wavelength.

### ROS/NO measurement

The intracellular ROS levels of cultured cells in 24-well plates were assessed by the use of an oxidation-sensitive fluorescent probe DCFH-DA (Eruslanov and Kusmartsev, 2010), and Rosup was included as a positive control (Beyotime, Shanghai, China). The intensity of DCF fluorescence was assessed by the use of a Lionheart FX Automated Microscope. NO production represented by nitrite concentration in the supernatants of cultured cells was assayed with Griess reaction in 96-well plates (Schulz et al., 1999) by the use of a NO Assay Kit (Beyotime, Shanghai, China). The absorbance was measured at 540 nm wavelength.

### Statistical analysis

Statistical analyses were conducted by the use of the GraphPad Prism 7.0 program (GraphPad Software).^1^ Data from triplicate wells (or more) from a representative of at least three independent experiments are presented as means ± SD. The statistical significance of the differences was assessed using Student’s t-test in comparison of two groups or one-way ANOVA in comparison of three or more groups. *p*-Values less than 0.05 were considered to be statistically significant (*p < 0.05, **p < 0.01).

## Data availability statement

The original contributions presented in the study are included in the article/Supplementary material, further inquiries can be directed to the corresponding author.

## Author contributions

ML and WL conceptualized, drafted, and revised the manuscript. ML, WZ, JW, and XY performed the experiments. ML and YY analyzed the data and elaborated the figures. All authors contributed to the article and approved the submitted version.

## Funding

This research was funded by the National Natural Science Foundation of China (32172885).

## Conflict of interest

The authors declare that the research was conducted in the absence of any commercial or financial relationships that could be construed as a potential conflict of interest.

## Publisher’s note

All claims expressed in this article are solely those of the authors and do not necessarily represent those of their affiliated organizations, or those of the publisher, the editors and the reviewers. Any product that may be evaluated in this article, or claim that may be made by its manufacturer, is not guaranteed or endorsed by the publisher.

## Footnotes

^1^ https://www.graphpad.com/

## Supplementary material

Supplementary Figure 1 *Giardia* induced HT-29 cell apoptosis.

Supplementary Table 1 Primer pairs used in qPCR analysis.
