# Corrigendum: Specific TLR-mediated HSP70 activation plays a potential role in host defense against the intestinal parasite *Giardia duodenalis*

**DOI:** 10.3389/fmicb.2024.1359801

**Published:** 2024-02-02

**Authors:** Min Liu, Yongwu Yang, Weining Zhu, Jingxue Wu, Xiran Yu, Wei Li

**Affiliations:** Heilongjiang Provincial Key Laboratory of Zoonosis, College of Veterinary Medicine, Northeast Agricultural University, Harbin, Heilongjiang, China

**Keywords:** *Giardia*, HSP70, host defense, apoptosis, nitric oxide, tight junction

In the published article, there was an error in Figure 2D as published. In the original Figure 2D, the Celastrol panels for HT-29 cell line were mistakenly placed. The corrected Figure 2D and its caption ^**^HSP70 regulated anti-apoptosis, cell survival, and NO levels. **(D)** HSP70-mediated regulation of cleaved CASP-3 as examined using immunofluorescence assay (scale bar = 100 μm) appear below.

**Figure 2:**
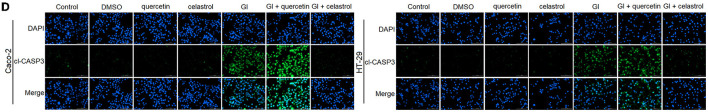
HSP70 regulated anti-apoptosis, cell survival, and NO levels. **(D)** HSP70-mediated regulation of cleaved CASP-3 as examined using immunofluorescence assay (scale bar = 100 μm).

The authors apologize for this error and state that this does not change the scientific conclusions of the article in any way. The original article has been updated.

