# Supplementary material for: Specific TLR-mediated HSP70 activation plays a potential role in host defense against the intestinal parasite Giardia duodenalis
Source: Front Microbiol. 2023 Mar 2;14:1120048. doi: 10.3389/fmicb.2023.1120048 (PMC10017776; doi:10.3389/fmicb.2023.1120048)
Supplement: Supplementary Figure 1 — Giardia induced HT-29 cell apoptosis. [file Image_1.PDF]

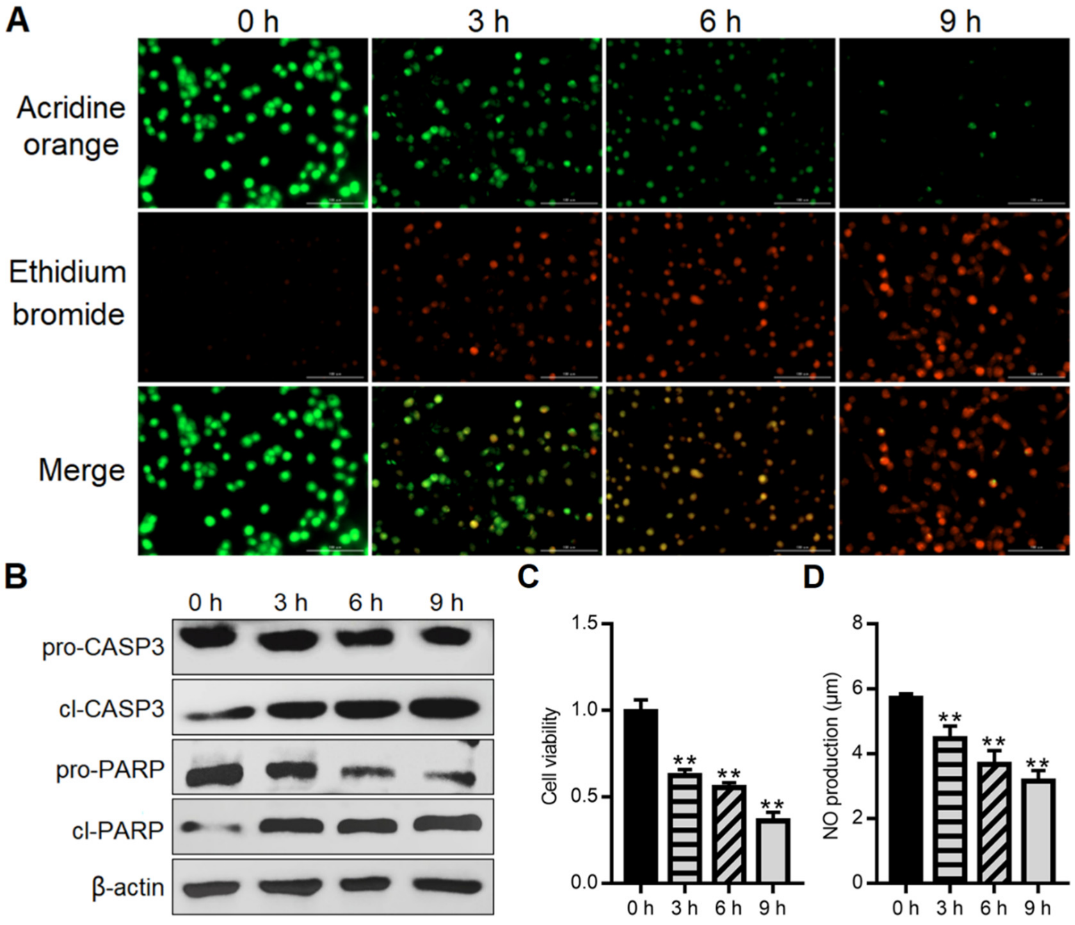

Supplementary Figure 1

Giardia induced HT-29 cell apoptosis. HT-29 cells were exposed to *Giardia* for 0, 3, 6, and 9 h. (A) *Giardia*-induced apoptosis as examined by AO/EB assay (scale bar = 100 μm). (B) *Giardia* infection enhanced the levels of cleaved CASP-3 and PARP as measured by western blot. (C) *Giardia* infection decreased the cell viability of HT-29 as measured by the CCK-8 assay. (D) *Giardia* infection decreased NO release as examined by a microplate reader. Data from triplicate wells from a representative of at least three independent experiments are presented as means ± SD. ** $P < 0.01$.
