# Supplementary material for: Specific TLR-mediated HSP70 activation plays a potential role in host defense against the intestinal parasite Giardia duodenalis
Source: Front Microbiol. 2023 Mar 2;14:1120048. doi: 10.3389/fmicb.2023.1120048 (PMC10017776; doi:10.3389/fmicb.2023.1120048)
Supplement: Supplementary Table 1 — Primer pairs used in qPCR analysis. [file Table_1.PDF]

Supplementary Table 1

Primer pairs used in qPCR analysis.

Gene	Accession no.	Primer (5′ to 3′)	Product size
HSP70	NM_002154.4	Forward: GCATCGAGACTATCGCTAATGAG<br>Reverse: TGCAAGGTTAGATTTTTCTGCCT	200 bp
HSP90	NM_005348.4	Forward: AGGAGGTTGAGACGTTCGC<br>Reverse: AGAGTTCGATCTTGTTTGTTCGG	223 bp
HSP27	NM_001540.5	Forward: ACGGTCAAGACCAAGGATGG<br>Reverse: AGCGTGTATTTCCGCGTGA	104 bp
CASP8	NM_001372051.1	Forward: TTTCTGCCTACAGGGTCATGC<br>Reverse: TGTCCAACTTTCCTTCTCCCA	183 bp
CASP9	NM_001229.5	Forward: CTCAGACCAGAGATTCGCAAAC<br>Reverse: GCATTTCCCCTCAAACTCTCAA	116 bp
CASP3	NM_004346.4	Forward: GAAATTGTGGAATTGATGCGTGA<br>Reverse: CTACAACGATCCCCTCTGAAAAA	164 bp
Apaf-1	NM_181869.2	Forward: AAGGTGGAGTACCACAGAGG<br>Reverse: TCCATGTATGGTGACCCATCC	116 bp
Bax	NM_138763.4	Forward: CCCGAGAGGTCTTTTTCCGAG<br>Reverse: CCAGCCCATGATGGTTCTGAT	155 bp
Bcl-2	XM_017025917.2	Forward: GGTGGGGTCATGTGTGTGG<br>Reverse: CGGTTCAGGTACTCAGTCATCC	89 bp
cIAP-1	NM_001256163.1	Forward: AGCACGATCTTGTCAGATTGG<br>Reverse: GGCGGGGAAAGTTGAATATGTA	102 bp
cIAP-2	NM_001165.5	Forward: TTTCCGTGGCTCTTATTCAAACT<br>Reverse: GCACAGTGGTAGGAACTTCTCAT	96 bp
RIP1	NM_001317061.3	Forward: TGGGCGTCATCATAGAGGAAG<br>Reverse: CGCCTTTTCCATGTAAGTAGCA	165 bp
ZO-1	NM_001330239.4	Forward: ACCAGTAAGTCGTCCTGATCC<br>Reverse: TCGGCCAAATCTTCTCACTCC	128 bp
Occludin	NM_002538.4	Forward: CGGCGAGTCCTGTGATGAG<br>Reverse: TCTTGTATTCCTGTAGGCCAGT	119 bp
GAPDH	NM_001357943.2	Forward: ACAACTTTGGTATCGTGGAAGG<br>Reverse: GCCATCACGCCACAGTTTC	101 bp
